# Acute combined effects of concurrent physical activities on autonomic nervous activation during cognitive tasks

**DOI:** 10.3389/fphys.2024.1340061

**Published:** 2024-02-19

**Authors:** Shan Cheng, Wenbin Li, Duoduo Hui, Jin Ma, Taihui Zhang, Chaolin Teng, Weitao Dang, Kaiwen Xiong, Wendong Hu, Lin Cong

**Affiliations:** ^1^ Department of Aerospace Medical Equipment, School of Aerospace Medicine, Air Force Medical University, Xi’an, Shaanxi, China; ^2^ Department of Aerospace Hygiene, School of Aerospace Medicine, Air Force Medical University, Xi’an, Shaanxi, China

**Keywords:** heart rate variability, cognitive task, physical load, combined effects, mental work scenarios, electrocardiogram (ECG)

## Abstract

**Backgrounds:** The validity of heart rate variability (HRV) has been substantiated in mental workload assessments. However, cognitive tasks often coincide with physical exertion in practical mental work, but their synergic effects on HRV remains insufficiently established. The study aims were to investigate the combined effects of cognitive and physical load on autonomic nerve functions.

**Methods:** Thirty-five healthy male subjects (aged 23.5 ± 3.3 years) were eligible and enrolled in the study. The subjects engaged in n-back cognitive tasks (1-back, 2-back, and 3-back) under three distinct physical conditions, involving isotonic contraction of the left upper limb with loads of 0 kg, 3 kg, and 5 kg. Electrocardiogram signals and cognitive task performance were recorded throughout the tasks, and post-task assessment of subjective experiences were conducted using the NASA-TLX scale.

**Results:** The execution of n-back tasks resulted in enhanced perceptions of task-load feelings and increased reaction times among subjects, accompanied by a decline in the accuracy rate (*p* < 0.05). These effects were synchronously intensified by the imposition of physical load. Comparative analysis with a no-physical-load scenario revealed significant alterations in the HRV of the subjects during the cognitive task under moderate and high physical conditions. The main features were a decreased power of the high frequency component (*p* < 0.05) and an increased low frequency component (*p* < 0.05), signifying an elevation in sympathetic activity. This physiological response manifested similarly at both moderate and high physical levels. In addition, a discernible linear correlation was observed between HRV and task-load feelings, as well as task performance under the influence of physical load (*p* < 0.05).

**Conclusion:** HRV can serve as a viable indicator for assessing mental workload in the context of physical activities, making it suitable for real-world mental work scenarios.

## 1 Introduction

In specific occupations such as airplane pilots and automobile drivers, mental workload constitutes a crucial risk factor that imperils personnel safety and demands careful consideration. Elevated mental load during tasks can precipitate mental fatigue, which eventually leads to an escalation in human errors and safety accidents (Jacquet et al., 2020; Ramírez-Moreno et al., 2021). Statistics indicate a direct correlation between driver fatigue and 20% of traffic accidents (Wilson et al., 2020). Conversely, insufficient mental workload may compromise alertness levels, hindering the timely mobilization of brain resources and potentially resulting in an accident. Real-time monitoring of the mental load level of operators during tasks holds practical significance, enabling the prompt and accurate identification of mental status and effectively minimizing the risk of accidents.

Current mental workload assessment methods, encompassing subjective and objective approaches, have distinct advantages and drawbacks. Subjective methods, such as the NASA task load index (NASA-TLX) (Wilson et al., 2021) and fatigue assessment scale (Ramírez-Moreno et al., 2021), use rating scales to evaluate mental workload at specific moments but face challenges in real-time monitoring due to questionnaire interruptions. Maintaining the natural working environment and operators’ activities is crucial, but subjective methods may compromise task execution (Albuquerque et al., 2020). In contrast, objective measurement methods, including electroencephalogram (EEG) (Zhang et al., 2021; Chitti et al., 2021; Wriessnegger et al., 2021), eye movement features (represented by blink, pupil diameter and saccade) (Bafna and Hansen, 2021), heart rate variability (HRV) (Burlacu et al., 2021) and postural stability indicators (Cheng et al., 2018; Sun et al., 2019), rely on biological signals and offer promising avenues for effective and objective mental workload assessment without disrupting tasks. These approaches pave the way for more precise and real-time monitoring, ensuring a comprehensive understanding of mental workload dynamics during various activities.

Recent progress in wearable technology has revolutionized the acquisition of biological signals in real work scenarios. Advanced features such as wireless transmission, miniaturized amplifiers (Wascher et al., 2023), and dry electrodes (Ramírez-Moreno et al., 2021) have enhanced the application of highly portable EEG head-mounted devices, proving crucial for research in real mobile environments. Technologies like the ballistocardiogram (BCG), using a fiber sensor cushion (Xu et al., 2021) or photoplethysmogram (PPG) integrated into a helmet (Wilson et al., 2020) have enabled the simultaneous collection of biological signals alongside the primary task, offering the capability to capture changes in the operators’ functional status before an alteration in task performance occurs. With superior temporal resolution compared to subjective methods, these approaches serve as pivotal tools for real-time mental workload assessment during tasks.

Comparing ECG to portable EEG acquisition technology, the former exhibits significantly enhanced anti-interference capabilities (Wang et al., 2016; Dai et al., 2021; Klug and Gramann, 2021; Teng et al., 2021), showing efficacy in evaluating mental fatigue within laboratory settings. Studies have demonstrated a correlation between ECG signals and psychomotor vigilance task (PVT) performance, as well as the effectiveness of HRV indices in assessing cognitive task-related errors. Leveraging sensitive features extracted from ECG, algorithms like learning vector quantization and random forest tree classifiers achieve impressive accuracy in identifying fatigue states, underscoring HRV as a potential indicator for evaluating worker fatigue (Chua et al., 2012; Zhao et al., 2012; Pan et al., 2021; Xu et al., 2021; Takada et al., 2022).

However, the authentic mental work environment, dominated by a cognitive load (CL), is frequently accompanied by physical activities, stress, pressure and other non-cognitive factors, all of which can alter the characteristics of biological signals. Currently, researchers have initiated investigations into the impact of increased physical load (PL), stress and other factors during cognitive tasks on physiological and psychological functions. For instance, significant changes in EEG-related indexes were observed when subjects engaged in a cognitive task while cycling (Xu et al., 2018). In the study by Zink and others, subjects were tasked with performing a three-level Oddball auditory task while cycling. EEG analysis showed a decrease in the P300 component of event-related potentials during physical activities in unrestrained conditions (Zink et al., 2016). Blons research group found that vagus nerve activities and entropy indexes increased in the cognitive task alone, while the entropy index of HRV decreased with the addition of stress factors (Blons et al., 2019). Confounding factors in a real work scenario may disrupt the balance of sympathetic and parasympathetic nerve activity, resulting in substantial variations in sensitivity (47.1%–95%) and specificity (74.6%–98%) in driving fatigue recognition parameters based on HRV indicators across different studies (Burlacu et al., 2021).

While mental workload in most existing research protocols is induced by sleep deprivation (Cheng et al., 2021), continuous cognitive tasks such as an n-back task (Karthikeyan et al., 2021; Wriessnegger et al., 2021) and a Stroop task (Nikooharf Salehi et al., 2022), these protocols often overlook the role of non-cognitive variables, particularly physical factors. The real-time evaluation of mental workload based on ECG signals is undoubtedly influenced by dynamics. Because the working process of mental workers is not static, then PL becomes an important non-cognitive factors. Therefore, the aims of this study were to explore the changes in ECG signals during different PL and CL under a background of dynamic tasks.

## 2 Materials and methods

### 2.1 Subjects

Thirty-five male subjects, aged between 20 and 32 years (mean 23.5 ± 3.3 years), were enrolled in the study. Inclusion criteria were: being right-handed; absence of diseases and injury affecting physical activities in the past 3 months; and no recent use of alcohol and specific medications. Based on self-reporting, subjects had a minimum of ≥6 h of sleep per day during the week preceding the study experiments. Additionally, subjects willingly provided written informed consent and agreed not to withdraw from the ongoing study for subjective reasons or concerns.

### 2.2 Experimental tasks

#### 2.2.1 Design of cognitive load

In the current study, the n-back working memory task program introduced by Wriessnegger et al. (2021), was applied to induce mental workload and fatigue states in the study subjects. At the initiation of the n-back task, subjects received instructions, followed by a sequence of letters. Their task was determined whether the current presented letter matched the one shown n letters prior, essentially identifying the target letter in the sequence. The criteria for the target letter were established such that if the current letter matched the one presented n letter before, it was considered the target letter. Three variation of n-back tasks (1-back, 2-back, and 3-back) with differing difficulty levels were employed. Each trial consisted of 20 letters, including 5 target letters. Prior to the trial, a 2-s instruction informed subjects about the type of n-back task to be performed. Subsequently, each trial lasted 40 s, with each letter displayed for 0.5 s and an interval of 1.5 s. Subjects were required to press the symbol “↑” symbol on the keyboard upon recognizing the target letter. Following the completion of a trial, subjects had a 6-s break. Thus, one trial encompassed 50 s. To complete each run of a specific n-back task type, a total of 6 consecutive trials were conducted, taking approximately 5 min.

#### 2.2.2 Design of physical load

In these experiments, isotonic contraction of the left upper limb was used to simulate the PL encountered during mental work, ensuring no interfere with n-back tasks performed using the right hand. Contractile resistance was introduced by holding a dumbbell, categorized into three levels of PL: no PL (none, 0 kg); moderate PL (medium, 3 kg); and a high PL (high, 5 kg). When applying PL to the subject, the preparation posture involved a 90-degrees flexion of the left arm. During lifting, the forearm was maintained an angle <45° from the upper arm, indicating effective contraction. The lift frequency during the cognitive task was once every 2 s, aligning with the letter presentation frequency of n-back tasks.

### 2.3 Assessment of task load

#### 2.3.1 Subjective scale assessment

The NASA-TLX was used to evaluate the experiences of subjects across six dimensions: mental demand, physical demand, time demand, self-performance, effort level and the frustration level. Subjects self-evaluated their feelings on a 21-point scale for each dimension. The six dimensions were combined into 15 pairs, and subjects were tasked with selecting the more significant factor within each pair. Subsequently, weights ranging from 1/21 to 6/21 were assigned based on the frequency of selections, ordered from the lowest to the highest. The total score was then calculated.

#### 2.3.2 HRV based on ECG data

In this study, the Dutch Spirit multi-channel biofeedback system was used to record ECG data at a sampling frequency of 256 Hz during the task. Initially, municipal electricity interference, myoelectric interference and baseline drift noise in ECG signal were mitigated using median filtering and moving average methods. Subsequently, the difference threshold method was used to extract the peak value of QRS, yielding the RR interval. To ensure the accuracy of RR interval calculation, outlier values were identified and screened using the fill outliers’ function in Matlab software, followed by linear interpolation between adjacent non-outlier values. This processed resulted in the acquisition of a preprocessed RR interval sequence from the raw ECG signal. The HRV characteristics of each stage were extracted based on the RR interval.

The time domain characteristic indices were computed through time series analysis of the RR interval, including: (1) mean heart rate (meanHR); (2) standard deviation of normal-to-normal beats (SDNN), representing the overall profile of HRV by calculating the standard deviation of all RR intervals within a specific time; (3) the number and percentage of difference between adjacent RR intervals exceeding 50 ms (NN50, pNN50), sensitive indicators of vagus nerve activity, with higher values signifying increased vagus nerve excitability; and (4) root mean square difference (RMSSD); the root mean square value of the differences between adjacent RR intervals, reflecting the high-frequency component of HRV, where higher values indicate greater vagal tone.

Autoregressive models were used in this study to analyze the frequency domain characteristics of HRV. The power spectrum of the RR interval sequence was categorized into three primary frequency bands: high frequency band (HF: 0.150–0.400 Hz), low frequency band (LF: 0.040–0.150 Hz), and very low frequency band (VLF: 0.003–0.040 Hz). Key frequency-domain parameters included: (1) VLF, LF and HF, representing the absolute power density in VLF, LF, and HF bands, respectively. LF indicates the level of sympathetic nerves activity, while HF reflects the regulatory strength of the vagus nerve. Total power density (Total) is the sum of VLF, LF and HF, providing a measure of overall HRV; (2) the LF/HF ratio, signifies the balance between sympathetic and vagal nerve activity; (3) the normalized low-frequency power (nLF) is the value of LF relative to the sum of LF and HF, indicating the relative levels of sympathetic nerves activity; and (4) normalized HF power (nHF), reflecting the degree of vagal regulation.

#### 2.3.3 Cognitive performance

The outcomes derived from the n-back task employed in this study primarily fall into two categories: (1) Reaction time (RT) for identifying the target letter, encompassing mean RT (MRT), standard deviation of RT (SDRT), maximum RT (maxRT), and minimum RT (minRT); (2) Performance indicators consisted of the ratio of correct reactions (CNR), the ratio of missing reactions (MNR) and ratio of incorrect reactions (WNR).

### 2.4 Experimental procedure

**FIGURE 1:**
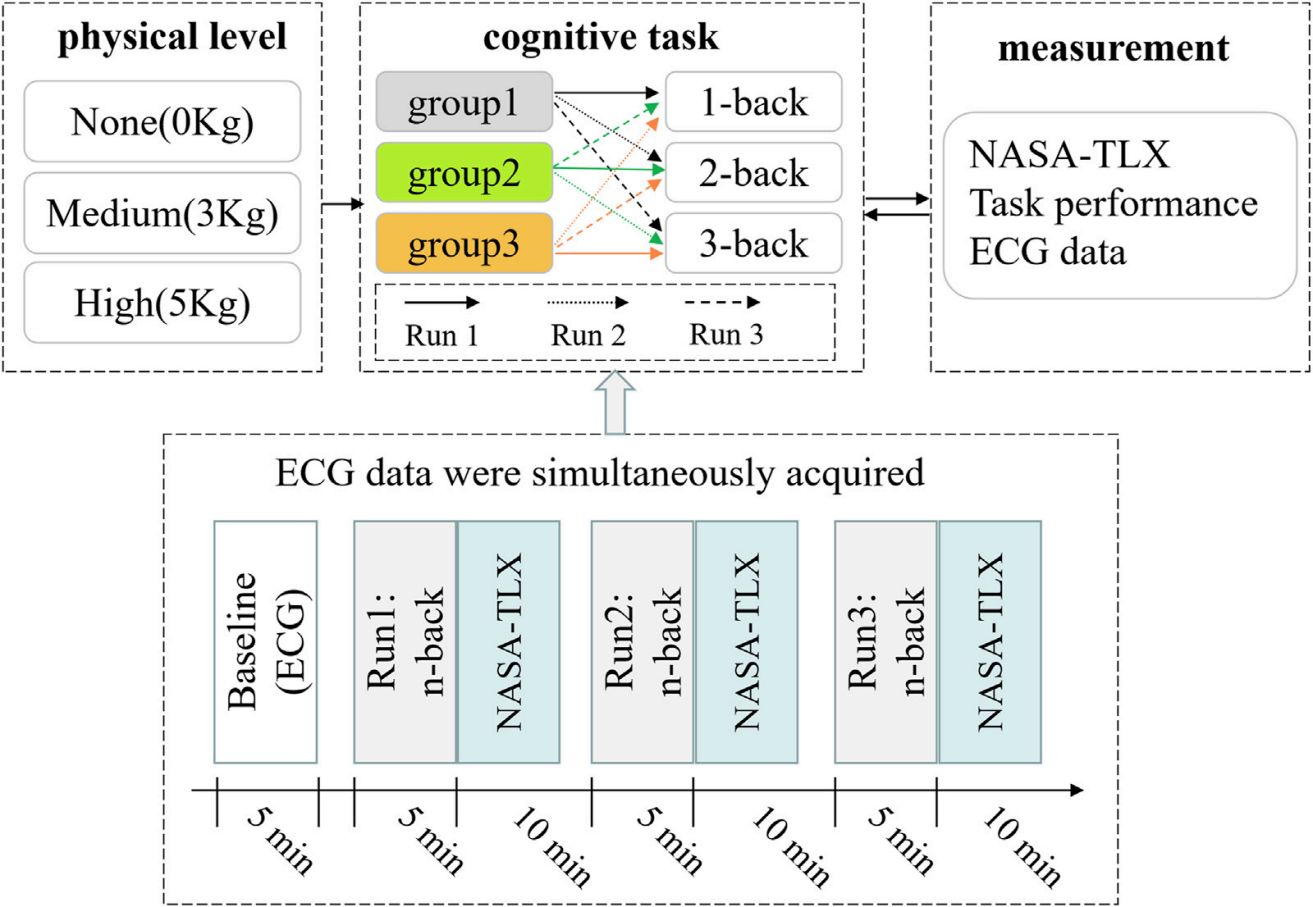
Experimental design and flow chart. Run1, Run2, and Run3 represent n-back tasks with three difficulty levels running in a preset order, respectively.

In this study, two independent variables (CL and PL) were established and implemented through a repeated measurement design with cross-control within the group (Figure 1). Specifically, CL featured three levels (1-back, 2-back, 3-back), while PL also had three levels (None, Medium, High). The experimental protocol unfolded across three stages, each with distinct PL levels, and there was a 1-week interval between stages. First, the ECG signals were collected from subjects in a resting state before each stage, serving as the baseline data. The three electrodes of the ECG were placed, respectively beneath the midpoint of the left clavicle, beneath the midpoint of the right clavicle, and slightly to the left of the xiphoid process.

Subsequently, subjects sequentially performed n-back cognitive tasks of varying difficulty levels, with concurrent recording of ECG signals. Following each run of n-back tasks, the NASA-TLX index was used to evaluate the subjective task load.

When conducting the n-back tasks across three difficulty levels (1-back, 2-back, 3-back), a crossover design within the group was implemented to avoid the effect of task orders (Figure 1). For example, under the no-physical level condition (none), all subjects were equitably and randomly allocated into three sub-groups (group 1, group 2, and group 3) to execute the n-back task following a pre-designed sequence. Group 1 underwent tasks in the order of 1-back, 2-back and 3-back; group 2 performed cognitive tasks in the sequence of 2-back, 3-back and 1-back; group 3 executed tasks in the order of 3-back, 1-back, and 2-back. A 10-min interval was allowed between successive n-back tasks. A similar task order protocol was applied to subjects in the medium and high physical level conditions, as in the no-physical condition.

### 2.5 Statistical analysis

All statistical analyses were performing using SPSS ver. 23.0. In cases where the collected data exhibited a normal distribution and homogeneity of variance, repeated measures analysis of variance (rmANOVA) and a paired *t*-test were used for multiple comparisons. If there were assumptions that were not met, the Friedman test and Wilcoxon signed rank test were employed. First, the analysis focused on examining differences of subjective feelings and task performance across n-back cognitive tasks to assess mental load levels. Subsequently, the impact of PL on HRV features were analyzed. Finally, Spearman’s rank correlation was used to evaluate the correlation between HRV and subjective feelings, as well as task performance. A significance of *p* < 0.05 was considered to indicative of a statistically significant finding.

## 3 Results

### 3.1 Mental load level induced by n-back tasks under PL

The main effects of CL and PL on NASA-TLX score and task performance were determined through rmANOVA. As the difficulty of the n-back task increased, the subjective task load demonstrated a significant rise (mental demand: *F = 44.800, p < 0.001;* effort: *F = 12.412, p < 0.001*; frustration level: *F = 24.350, p < 0.001*). Additionally, an increase in PL resulted in a significant elevation of the task load level, particularly in time demand (*F = 5.026, p = 0.007*) and effort (*F = 11.078, p < 0.001*). Similarly, the cognitive tasks had a significant impact on subjects’ reaction time, leading to a significant prolongation (MRT: *F = 58.045, p < 0.001*; SDRT: *F = 90.316, p < 0.001*), along with a remarkable decrease in the rate of correct responses (CNR: *F = 73.439, p < 0.001*). PL also influenced subjects’ reaction times, resulting in a significant prolongation (MRT: *F = 3.924, p = 0.021;* SDRT: *F = 4.770, p = 0.009*) and a decrease in CNR (*F = 3.984, p = 0.020*) (See Supplementary Material).

### 3.2 Combined effects of PL with CL on autonomic nervous activity

**FIGURE 2:**
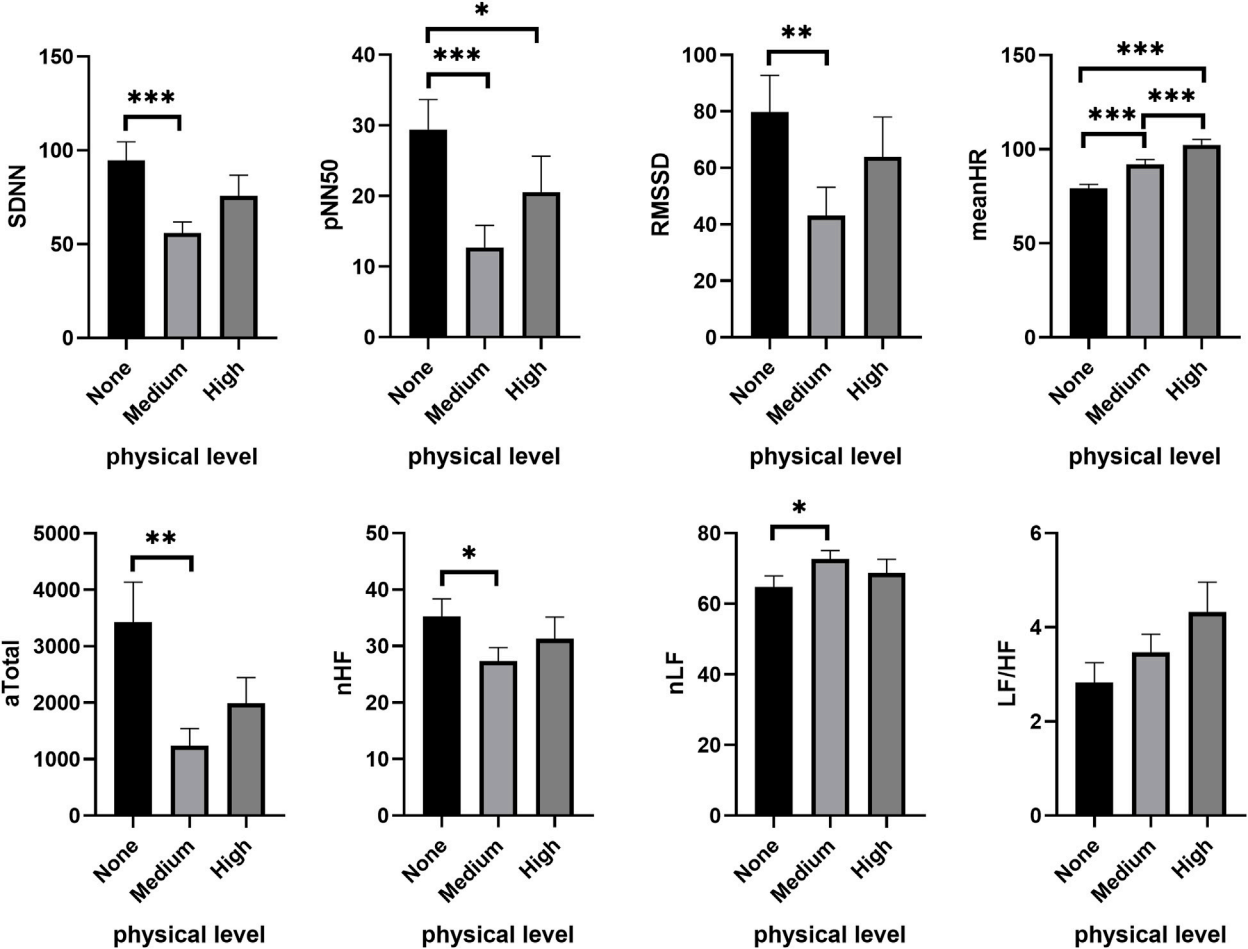
Changes of HRV features under different PL in the low CL condition. “None,” “Medium” and “High” respectively show the physical level during tasks. *p < 0.05, **p < 0.01, ***p < 0.001. Data are plotted the mean ± SEM. aTotal, Absolute value of total power density; CL, cognitive load; LF/HF, ratio of low-frequency and high frequency power; meanHR, mean heart rate; nHF, normalized high frequency power; nLF, normalized low-frequency power; PL, physical load; pNN50, percentage of the difference between adjacent RR intervals >50 ms; RMSSD, root mean square difference; SDNN, standard deviation of normal to normal beats.

The autonomic nervous system exhibited similar activity across different PL groups (See Supplementary Material). Under a low CL condition (1-back), PL significantly reduced SDNN (*χ* ^*2*^ = 21.063, *p* < 0.001), NN50 (*χ* ^*2*^ = 12.110, *p* = 0.002), pNN50 (*χ* ^*2*^ = 17.055, *p* < 0.001) and RMSSD (*χ* ^*2*^ = 12.000, *p* = 0.002), while meanHR obviously increased (*χ* ^*2*^ = 40.268, *p* < 0.001). In comparison to no PL (none), subjects’ SDNN (Wilcoxon signed ranks test, *Z* = −4.282, *p* < 0.001), NN50 (*Z* = −3.413, *p* = 0.001), pNN50 (*Z* = −3.805, *p* < 0.001), RMSSD (*Z* = −3.272, *p* = 0.001) and meanHR (*Z* = −3.572, *p* < 0.001) were all significantly increased at medium PL (Figure 2). At high PL, the subjects’ pNN50 (*Z* = −2.281, *p* = 0.023) was significantly decreased, while meanHR (*Z* = −4.544, *p* < 0.001) significantly increased. Compared with medium PL, meanHR (*Z* = −3.331, *p* < 0.001) significantly increased. In addition, PL significantly reduced the power density value of HRV, including VLF (*χ* ^*2*^ = 21.438, *p* < 0.001), LF (*χ* ^*2*^ = 10.938, *p* = 0.004), HF (*χ* ^*2*^ = 9.750, *p* = 0.008) and Total (*χ* ^*2*^ = 10.563, *p* = 0.005), all of which decreased significantly. Compared to no PL, VLF (*Z* = −3.160, *p* = 0.002), LF (*Z* = −3.160, *p* = 0.002), HF (*Z* = −3.141, *p* = 0.002), Total (*Z* = −3.403, *p* = 0.001), nHF (*Z* = −2.151, *p* = 0.032) decreased significantly at medium PL, while nLF (*Z* = −2.151, *p* = 0.032) increased significantly. At high PL, VLF (*Z* = −4.245, *p* < 0.001) was further reduced.

**FIGURE 3:**
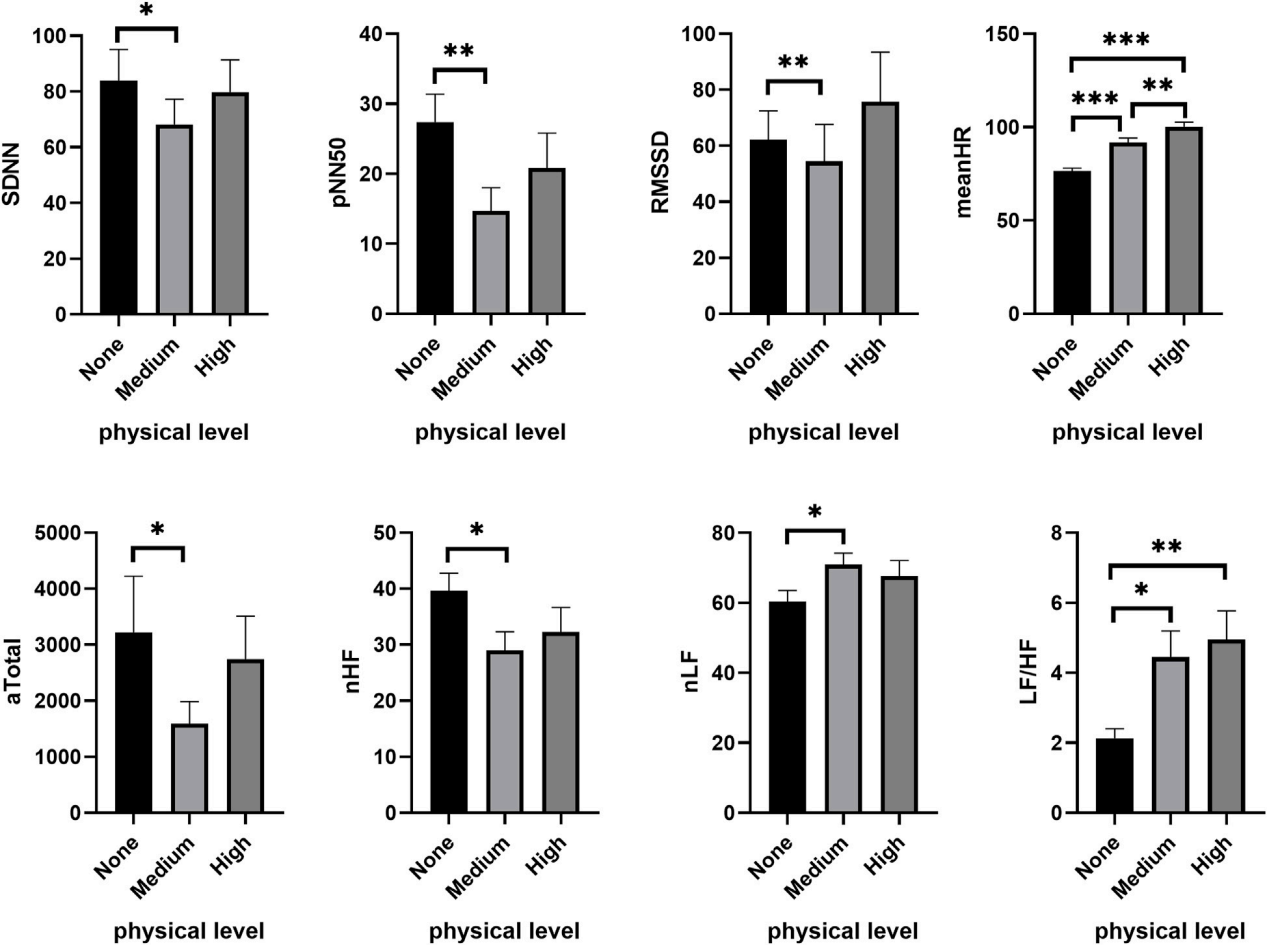
Changes of HRV features under different PL in the moderate CL condition. *p < 0.05, **p < 0.01, ***p < 0.001. Data are plotted as the mean ± SEM. CL, cognitive load; PL, physical load.

The effects of PL on HRV under higher CL (2-back and 3-back) mirrored that observed under low CL (1-back). In the moderate CL condition (2-back), PL significantly decreased SDNN (*χ* ^*2*^ = 6.750, *p* = 0.034), NN50 (*χ* ^*2*^ = 14.736, *p* = 0.001), pNN50 (*χ* ^*2*^ = 16.528, *p* < 0.001) and RMSSD (*χ* ^*2*^ = 11.313, *p* = 0.003), while the meanHR increased (*χ* ^*2*^ = 43.938, *p* < 0.001). Furthermore, PL also significantly reduced the absolute values of HF (*χ* ^*2*^ = 12.000, *p* = 0.002) and Total (*χ* ^*2*^ = 7.000, *p* = 0.030) during cognitive tasks. As shown in Figure 3, compared to no PL (none), time-domain and frequency-domain features of HRV underwent significant changes under the medium PL condition, including SDNN (*Z* = −2.122, *p* = 0.034), NN50 (*Z* = −3.245, *p* = 0.001), pNN50 (*Z* = −3.282, *p* = 0.001), RMSSD (*Z =* −2.655, *p* = 0.008), LF/HF (*Z* = −2.580, *p* = 0.010), and the proportion of low frequencies (nLF) (*Z* = −2.188, *p* = 0.029). These latter changes in HRV were not obvious under the high PL condition.

**FIGURE 4:**
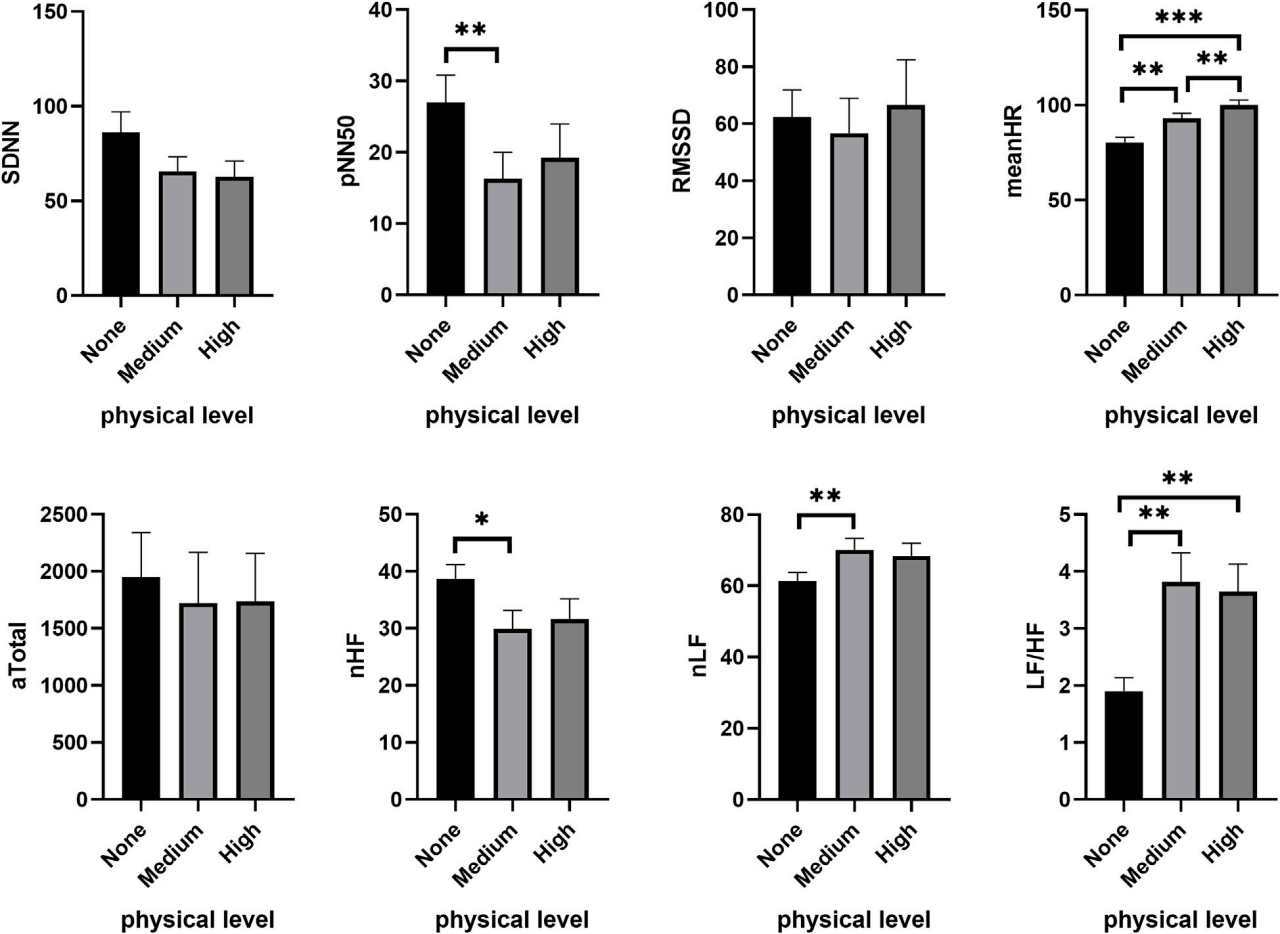
Changes of HRV features under different PL in high CL condition. *p < 0.05, **p < 0.01, ***p < 0.001. Data are presented as the mean ± SEM.

In the high CL condition (3-back), subjects exhibited a significant decrease in SDNN (*χ* ^*2*^ = 6.063, *p* = 0.048), NN50 (*χ* ^*2*^ = 8.368, *p* = 0.015), pNN50 *(χ* ^*2*^ = 9.424, *p* = 0.009) and RMSSD (*χ* ^*2*^ = 7.313, *p* = 0.026) with increasing PL, along with a significant rise in meanHR (*χ* ^*2*^ = 26.313, *p* < 0.001). The reduction in subjects’ HF (*χ* ^*2*^ = 7.938, *p* = 0.019), Total (*χ* ^*2*^ = 6.750, *p* = 0.034), nHF (*χ* ^*2*^ = 6.813, *p* = 0.033), and the increase in the ratio of LF/HF (*χ* ^*2*^ = 9.250, *p* = 0.010) and nLF (*χ* ^*2*^ = 6.813, *p* = 0.033) were induced by increasing PL during the cognitive task. Figure 4 shows that certain HRV parameters changed noticeably at medium PL, including a decrease in NN50 (*Z =* −2.469, *p* = 0.014), pNN50 (*Z* = −2.739, *p* = 0.006) and the proportion of HF (nHF: *Z* = −2.394, *p* = 0.017), coupled with an increase in the LF/HF ratio (*Z* = −3.216, *p* = 0.001) and nLF (*Z* = −2.394, *p* = 0.017) (Figure 4). No significant difference was found between medium and high PL.

Regardless of the CL, these results suggested that subjects’ HR increased with an elevation in PL, while the absolute energy in different frequency bands decreased. The relative proportion of the frequency value increased, while the proportion of HF decreased.

### 3.3 Correlation of HRV with CL under the effects of PL

Spearman’s correlation analysis revealed a significant relationship between HRV indexes under different CL conditions and subjective feelings in the absence of PL (none). In Table 1, the NASA-TLX total score exhibited a significant positive correlation with the LF/HF ratio (*r* = 0.204, *p* = 0.049) and nLF (*r* = 0.226, *p* = 0.003), and a negative correlation with aTotal (*r* = −0.272, *p* = 0.008), nHF (*r* = −0.226, *p* = 0.003), SDNN (*r* = −0.324, *p* = 0.002), pNN50 (*r* = −0.260, *p* = 0.012) and RMSSD (*r* = −0.297, *p* = 0.004). Under the medium PL condition, the meanHR showed a positive correlation with the NASA-TLX score (*r* = 0.210, *p* = 0.044). Furthermore, the RT for a cognitive task exhibited a positive correlation with the LF/HF ratio (*r* = 0.223, *p* = 0.032) and nLF (*r* = 0.205, *p* = 0.049), while showing a negative correlation with nHF (*r* = −0.205, *p* = 0.049). In the presence of high PL, significant correlations were found between HRV with subjective workload. For example, the NASA-TLX total score revealed a significant correlation between LF/HF (*r* = 0.271, *p* = 0.009), nLF (*r* = 0.270, *p* = 0.009) and nHF (*r* = −0.270, *p* = 0.009).

**TABLE 1:** Correlation coefficients between HRV and cognitive task load under the influence of PL.

HRV	None physical level		Medium physical level		High physical level	
	Subjective feelings	Mean reaction time	Subjective feelings	Mean reaction time	Subjective feelings	Mean reaction time
aVLF	−.275**	−0.198	−0.042	0.028	−0.035	−0.042
aLF	−.240*	−0.071	−0.076	0.136	−0.069	−0.127
aHF	−.329**	−0.016	−0.082	0.035	−0.182	−0.116
aTotal	−.272**	−0.105	−0.053	0.08	−0.122	−0.095
LF/HF	.204*	−0.100	−0.013	.223*	.271**	0.028
nHF	−.226*	0.092	0.004	−.205*	−.270**	−0.027
nLF	.226*	−0.092	−0.004	.205*	.270**	0.027
SDNN	−.324**	−0.15	−0.023	0.095	−0.142	−0.146
NN50	−.228*	−0.031	0.004	0.100	−0.171	−0.086
pNN50	−.260*	−0.001	−0.034	0.08	−0.162	−0.087
RMSSD	−.297**	−0.045	−0.038	0.062	−0.156	−0.107
MeanHR	0.103	0.138	.210*	−0.049	−0.121	0.022

Note: Spearman’s rho was used to determine the correlation between HRV, and task load. *p < 0.05, **p < 0.01.

aHF, absolute value of power density in high frequency band; aLF, absolute value of power density in low frequency band; aTotal, absolute value of total power density; aVLF, absolute value of power density in very low frequency band; LF/HF, ratio of low-frequency and high frequency power; meanHR, mean heart rate; nHF, normalized high frequency power; nLF, normalized low-frequency power; NN50, number and percentage of the difference between adjacent RR, intervals >50 ms; pNN50, percentage of the difference between adjacent RR, intervals >50 ms; RMSSD, root mean square difference; SDNN, standard deviation of normal to normal beats.

## 4 Discussion

In the present study, the HRV of subjects were investigated during cognitive tasks under moderate and high physical conditions, and the results showed significant alterations compared to scenarios without physical load. Additionally, a clear linear correlation was found between HRV and task-load perceptions, as well as task performance under the influence of physical load (*p* < 0.05).

Researchers could approach the real-time monitoring of mental workload during dynamic tasks by considering the interaction between various physiological loads and cognitive tasks. Previous experimental paradigms employed to induce mental workload often overlooked the impact of non-cognitive factors (PL, stress, etc.) within an authentic dynamic work scenario. Cheng et al. investigated postural control changes in a mentally fatigue state induced by 36 h of sleep deprivation (Cheng et al., 2018). Karthikeyan et al. employed a 1-h visuo-spatial 2-back task to study the effects of anodal transcranial direct current stimulation on working memory under fatigue (Karthikeyan et al., 2021). Additionally, a 60 min Stoop color-word task (Nikooharf Salehi et al., 2022) and a flight simulator task (Pan et al., 2021) were used to induce a mental state of fatigue. While these models may be suitable for scenarios involving static operators, they might not be applicable to actual dynamic settings, such as those encountered by pilots, firefighters and first responders. Therefore, in the present study, isotonic contraction of the upper limbs of subjects at various resistances were used to simulate the PL that dynamic operators encounter during mental work.

Regardless of the impact of PL, the n-back tasks of varying difficulties in this study elicited distinct levels of mental load. Evaluation through the NASA-TLX scale showed significant increases in psychological needs, time demand, effort and frustration levels, coupled with a significant decrease in self-performance. The n-back task performance exhibited adverse effects, including the extension of the MRT and maxRT, an increase in wrong responding rates, and a decrease in correct rates. These outcomes are in good agreement with the findings of previous study (Wriessnegger et al., 2021). To induce diverse levels of mental workload, Wriessnegger et al. instructed subjects to engage in three types of n-back tasks (1-back, 2-back, and 3-back) for three trials (20 min each, 60 min in total) (Wriessnegger et al., 2021). Moreover, this study also found that PL could further enhance the subjective perceptions on task loads when subjects undertook n-back cognitive tasks. This result is consistent with previous outcomes, as demonstrated in the study by Albuquerque et al., where the mental demand scores of NASA-TLX further increased when subjects performed multi-cognitive tasks under the effect of PLs (Albuquerque et al., 2020).

Although our findings, indicating that PL can significantly increase the RT of cognitive tasks and reduce task performance, may appear somewhat inconsistent with previous studies, it is noteworthy that beneficial effects of physical activities have been reported. Most previous studies have demonstrated significantly faster response times during cognitive tasks (Zhu et al., 2021; Engeroff et al., 2022) and improved accuracy rates (Gao et al., 2021; Zheng et al., 2022) following physical activities or exercise interventions, such as cycling. Upon analyzing the experimental conditions, it became evident that the positive effects on works’ cognitive ability typically manifest after acute physical exercises, a condition distinct from our study.

The concurrent presence of PL has been associated with a reduction in EEG activity (Xu et al., 2018) and ERP component amplitudes (P_300_ wave) (Zink et al., 2016) during the performance of cognitive tasks. Studies utilizing functional near infrared spectroscopy have also suggested that while acute aerobic exercise improves working memory performance, activities in the bilateral frontal pole area, dorsolateral prefrontal cortex (dlPFC) and other regions may decrease (Zheng et al., 2022). However, after acute exercise, the degree of activation of dlPFC (Fujihara et al., 2021) and its correlation with behavioral performance were enhanced (Zhang et al., 2021). Taken together, these results suggest that improvement of behavioral performance after physical activity may stem from the compensatory activation of relevant brain areas after exercise.

In the absence of influence of PL, subjects’ HRV features underwent significant changes as the cognitive task load increased. The autonomic nervous system, consisting of the sympathetic and parasympathetic nervous systems, regulates the arousal level of workers and is closely related to fluctuation in HR (Maiorana et al., 2022; Garis et al., 2023). The substantial reduction in the high-frequency value of HRV and the low-frequency value may be indicative of increased activity in the sympathetic nervous system, and *vice versa*. Studies have reported that an increase in mental workload or the onset of mental fatigue leads to activation of the sympathetic nervous system (Wehrens et al., 2012; Chen et al., 2013). However, it is essential to acknowledge that physical activity has a significant influence on the autonomic nervous system. Alfonso et al. conducted a study involving 123 HRV recordings in the morning and 66 recordings of training intensity over a 6-week period with 5 recreational road cyclists. The results indicated that the higher training intensity on a given day correlated with a lower normalized HF and higher LF/HF values the next morning (Alfonso and Capdevila, 2022). It is plausible that under the influence of PL, sympathetic nervous system activity increases and vagal activity decreases.

Under the combined condition of a cognitive task and physical factors in our study, HRV also exhibited significant changes with an increase in PL, wherein the HF component of HRV decreased, and the LF component increased. These results suggest a decrease in vagal activity and an increase in sympathetic nervous system activity, aligning with observations made by previous researchers. Dallaway et al. studied the effects of mental fatigue on grip strength training by using a Stroop color classification task (with response inhibition) and n-back memory update task (without response inhibition). They found that the Stroop and 2-back tasks elicited higher HRs, lower HRV and greater fatigue compared to the control task (Dallaway et al., 2022). During isometric contraction of the quadriceps femoris while performing cognitive tasks, subjects’ sympathetic nervous system activity increased along with the sense of muscle exertion (Chatain et al., 2019). However, in the presence of PL, HRV did not exhibit statistically significant changes when subjects performed cognitive tasks at different levels. Another study that explored the cognitive task induced by smartphone use during resistance exercise found no significant effect on HRV (Fortes et al., 2022). These findings suggest that physical factors may play a dominant role in the combined effect, thereby attenuating the influence of CL on HRV.

Moreover, correlation analysis showed a liner relationship between HRV indexes, subjective feelings, and cognitive performance under the influence of PL during cognitive tasks. The degree of correlation varied in our study with different levels of PL. These results suggest that HRV remains a potential effective indicator of task load in actual dynamic mental work scenarios when combined with physical factors. Previous research also noted an increase in the high-frequency component of HRV and enhanced vagal activity when fatigue reached a critical level (Vaara et al., 2009; Konishi et al., 2013). Thus, as the degree of mental fatigue intensifies, the effects of CL and PL may be opposing, potentially compromising the evaluation validity of mental workload and fatigue states based on HRV.

One limitation of the present study was the use of a repeated-measures design to examine the primary effects of PL during cognitive tasks. A within-subjects experimental paradigm was applied when performing the n-back task under different PL conditions to cancel the influence of individual differences. To eliminate practicing effects, a 1-week interval between conditioning sessions was implemented; however, the PL condition was introduced later, potentially inducing an adaptation phenomenon. This study did not explore the validity of mental workload assessment based on ECG signals under the combined effects of PL and CL. Receiver operating curve analysis has indicated a good validity for fatigue assessment models, even when considering the influence of PL. Assessment models constructed by a random forest classifier showed average area under the curve values exceeding 0.995 for the two-classification mental workload method, based on features such as EEG, skin temperature, galvanic skin response and blood volume pulse features (Albuquerque et al., 2020). Further studies are needed to investigate the effect of physical factors on ECG-based evaluation models and their validity in assessing mental workload.

## 5 Conclusion

This study adopted the combined experimental paradigm involving CL and PL to simulate the characteristics of real dynamic mental work scenarios. The observed increase in subjects’ task-load perceptions and reaction times indicated an increased level of mental workload. Concurrent PL during cognitive tasks enhanced the reaction. Regarding the combined effects of PL and CL on autonomic nervous system activity, PL emerged as the dominant factor, marked by an elevation in the LF component and a reduction in the HF component of HRV features, with sympathetic nervous system excitation contributing to this phenomenon. The combined effect adds complexity to the real-time assessment and evaluation of mental work based on the ECG. Additionally, the findings highlighted that HRV can effectively reflect changes in subjective feelings and task performance during mental work under the effects of PL. It should be noted that the validity of HRV under more demanding mental working conditions requires further exploration. Our study offers preliminary evidence of the evolving features of HRV under a mental workload, laying a theoretical understanding for the accurate and real-time assessment of the task-load level for mental workers in authentic dynamic scenarios.

## Data Availability

The original contributions presented in the study are included in the article/Supplementary Material, further inquiries can be directed to the corresponding authors.
