# Supplementary material for: Acute combined effects of concurrent physical activities on autonomic nervous activation during cognitive tasks
Source: Front Physiol. 2024 Feb 19;15:1340061. doi: 10.3389/fphys.2024.1340061 (PMC10909997; doi:10.3389/fphys.2024.1340061)
Supplement: Supplementary file 1 [file DataSheet1.docx]

Supplementary Material

**1 Mental load level induced by n-back tasks under physical load**

**1.1 Changes of subjective feelings**

The main effects of cognitive load (CL) and physical load (PL) on NASA task load index (NASA-TLX) score were analyzed by repeated measures analysis of variance (rmANOVA). The analysis revealed that with increasing n-back task difficulty, mental demand (*F* = 44.800, *P* < 0.001), time demand (*F* = 22.546, *P* = 0.007), effort (*F* = 12.412, *P* < 0.001) and the frustration level (*F* = 24.350, *P* < 0.001) all increased, while self-performance significantly decreased (*F* = 15.061, *P* < 0.001). Under the influence of PL, the physical demands (*F* = 293.198, *P* < 0.001), time demand (*F* = 5.026, *P* = 0.007), effort (*F* = 11.078, *P* < 0.001), and frustration (*F* = 10.743, *P* < 0.001) all were significantly increased. As shown in **Figure S1**, when PL was constant, the increase of CL, mental demand, time demand, effort and frustration levels all were significantly increased, while self-performance was significantly decreased. The results of the analysis showed that regardless of PL, n-back tasks with different difficulties still induced different levels of mental load.


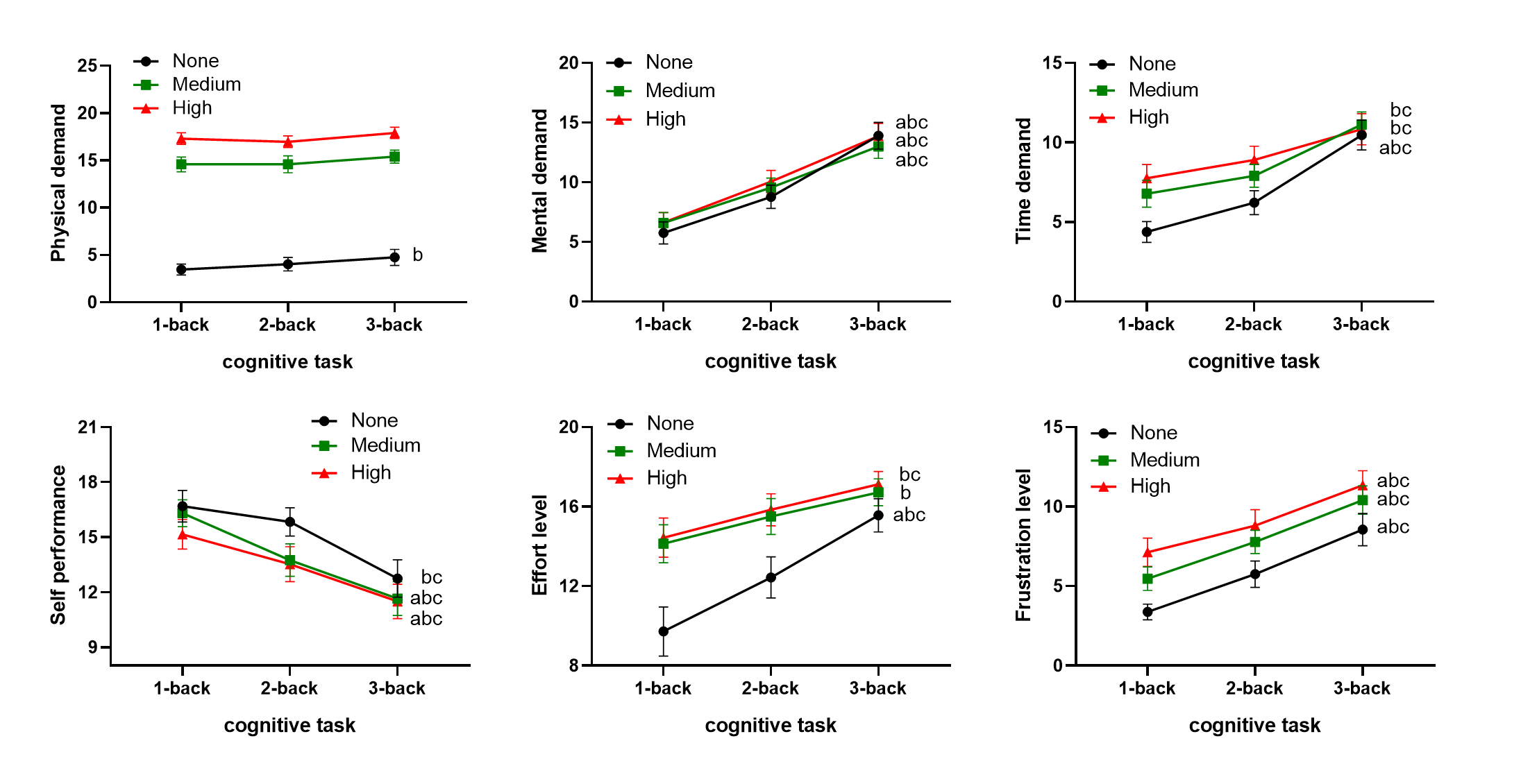


**FIGURE S1**

Effects of cognitive task on NASA-TLX results under PL. Repeated measures analysis of variance and a paired *t*-test were used. “a” represents the comparison between 1-back and 2-back, *P* < 0.05; “b” represents “*P* < 0.05” between 1-back and 3-back tasks; “c” represents “*P* < 0.05” between 2-back and 3-back. “None”, “Medium” and “High”, respectively show the physical level during the tasks. Data are plotted as the mean ± standard error. The sample size was 33. CL, cognitive load; NASA-TLX, NASA task load index; PL, physical load.

**1.2 Changes of cognitive tasks performance**

Under effect of cognitive tasks, subjects’ reaction time (RT) were significantly prolonged, such as mean RT (MRT) (*F* = 58.045, *P* < 0.001), standard deviation of RT (SDRT) (*F* = 90.316, *P* < 0.001) and maximum RT (maxRT) (*F* = 80.336, *P* < 0.001). And the ratio of correct reactions (CNR) of subjects (*F* = 73.439, *P* < 0.001) was significantly decreased, while the ratio of missing reactions (MNR) (*F* = 73.439, *P* < 0.001) and ratio of incorrect reactions (WNR) (*F* = 43.525, *P* < 0.001) were significantly increased. Similarly, under the influence of PL, MRT (*F* = 3.924, *P* = 0.021), SDRT (*F* = 4.770, *P* = 0.009) and maxRT (*F* = 6.432, *P* = 0.002) of the subjects were significantly prolonged. CNR (*F* = 3.984, *P* = 0.020) was significantly decreased, while MNR (*F* = 3.984, *P* = 0.020) and WNR (*F* = 3.901, *P* = 0.021) were both significantly increased.

In none PL condition (None), cognitive tasks significantly increased subjects’ MRT (*F* = 26.632, *P* < 0.001), SDRT (*F* = 35.119, *P* < 0.001) and maxRT (*F* = 35.747, *P* < 0.001). Under medium PL the subjects’ CL also increased MRT (*F* = 30.836, *P* < 0.001), SDRT (*F* = 32.126, *P* < 0.001) and maxRT (*F* = 36.874, *P* < 0.001). Under the influence of high PL (High), CL also significantly increased MRT, SDRT and maxRT (**Fig. S2**). For the performance indicators of cognitive tasks, regardless of PL, CNR was significantly decreased, while MNR and WNR were significantly increased with an increase in CL (**Fig. S3**).


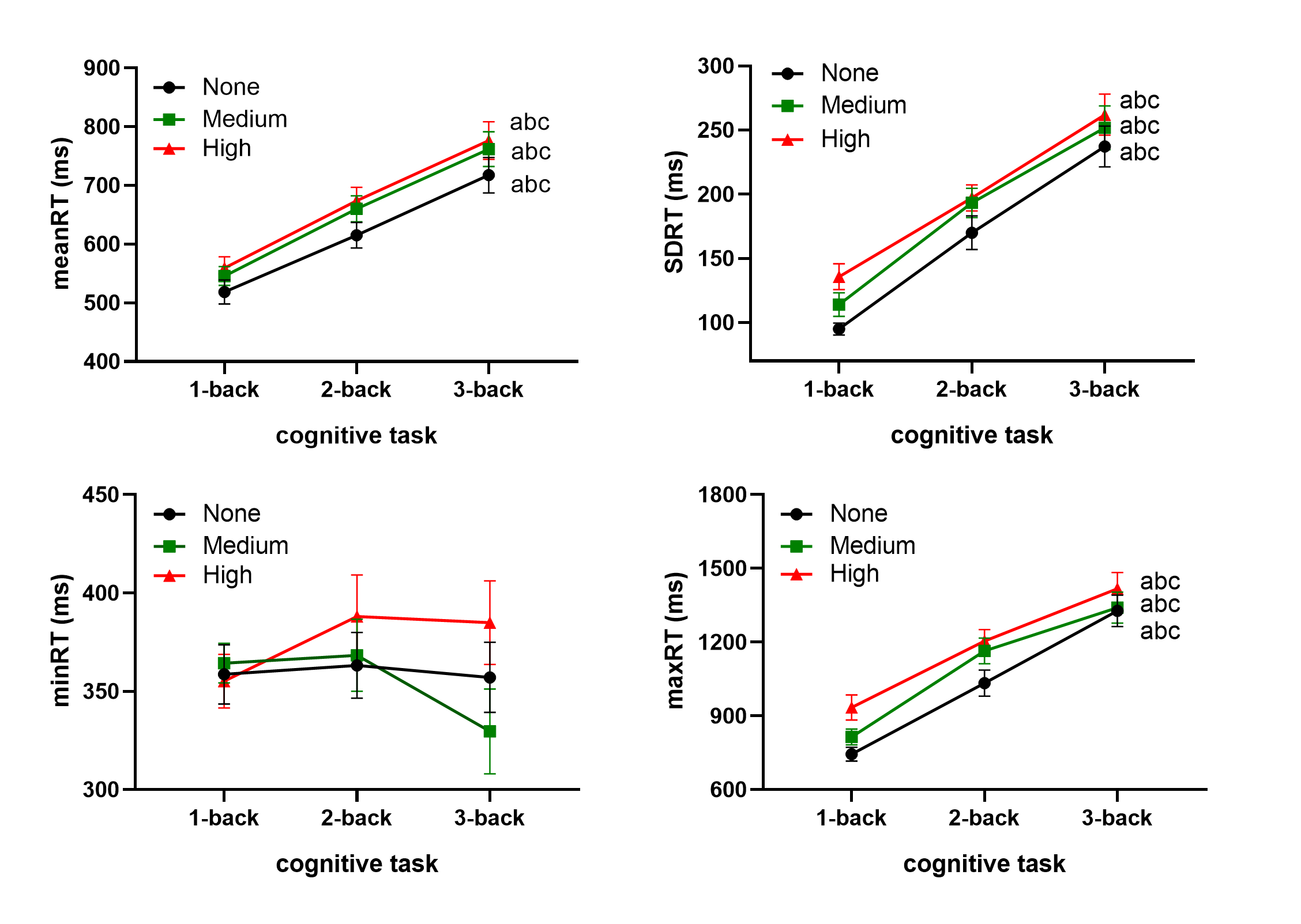


**FIGURE S2**

Changes of RT during n-back tasks. Repeated measures analysis of variance and a paired *t*-test were the statistical tests employed. The meanings of symbols (a, b and c) are the same as in **Figure S1**. maxRT, maximum reaction time; meanRT, average of reaction time; minRT, minimum reaction time; SDRT, standard deviation of reaction time.


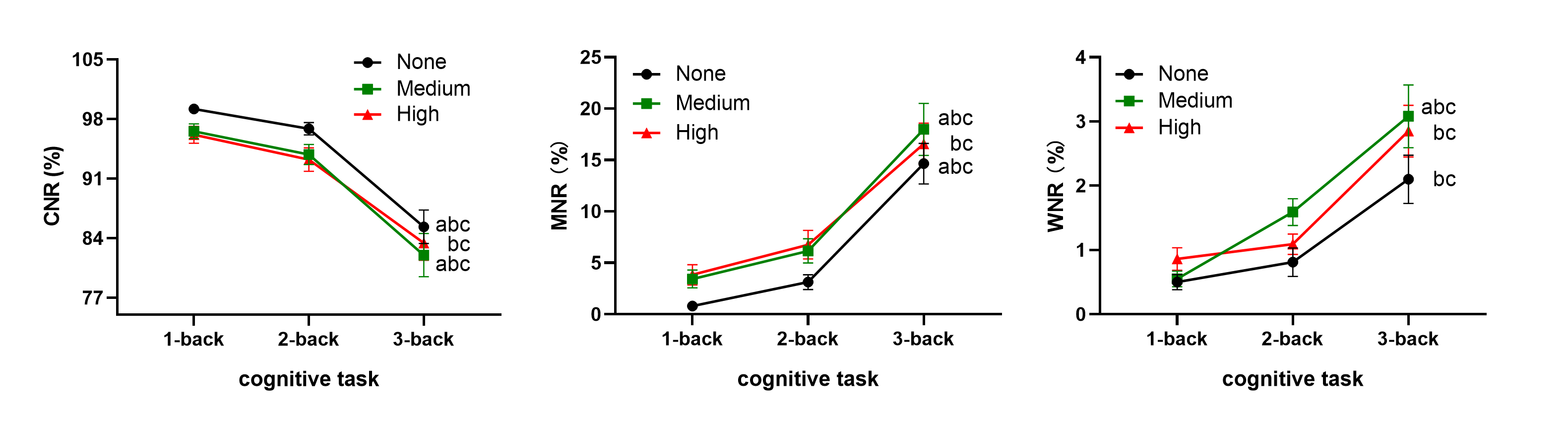


**FIGURE S3**

Changes of task performance during n-back tasks. Because CNR, MNR and WNR data did not conform to a normal distribution and homogeneity of variance, the Friedman test and Wilcoxon signed rank tests were used. The meaning of the symbols is the same as in **Figure S1**. CNR, the ratio of correct reactions; MNR, the ratio of missing reactions; WNR, the ratio of incorrect reactions.

**1.3 Effects of different CL on heart rate variability**

In these experiments, 3 subjects were excluded due to ECG electrode shedding leaving 32 subjects with valid data. Analysis of variance on the heart rate variability (HRV) parameters showed that there was no significant interaction between PL and CL. In the absence of physical factors, the change of CL had a significant effect on the absolute value of power density in very low frequency band (aVLF) (*χ^2^* = 10.938, *P* = 0.004) and absolute value of total power density (aTotal) (*χ^2^* = 8.063, *P* = 0.018). As shown in **Figure S4**, compared with low CL, aVLF (*Z* = -2.917, *P* = 0.004), absolute value of power density in low frequency band (aLF) (*Z* = -2.375, *P* = 0.018), aTotal (*Z* = -2.711, *P* = 0.007) and ratio of low-frequency and high frequency power (LF/HF) (*Z* = -2.057, *P* = 0.040) all significantly decreased under high CL. However, under moderate and high PL, the changes of CL had no significant effect on HRV. The results suggest that physical workload may alter the changes of HRV during cognitive tasks.


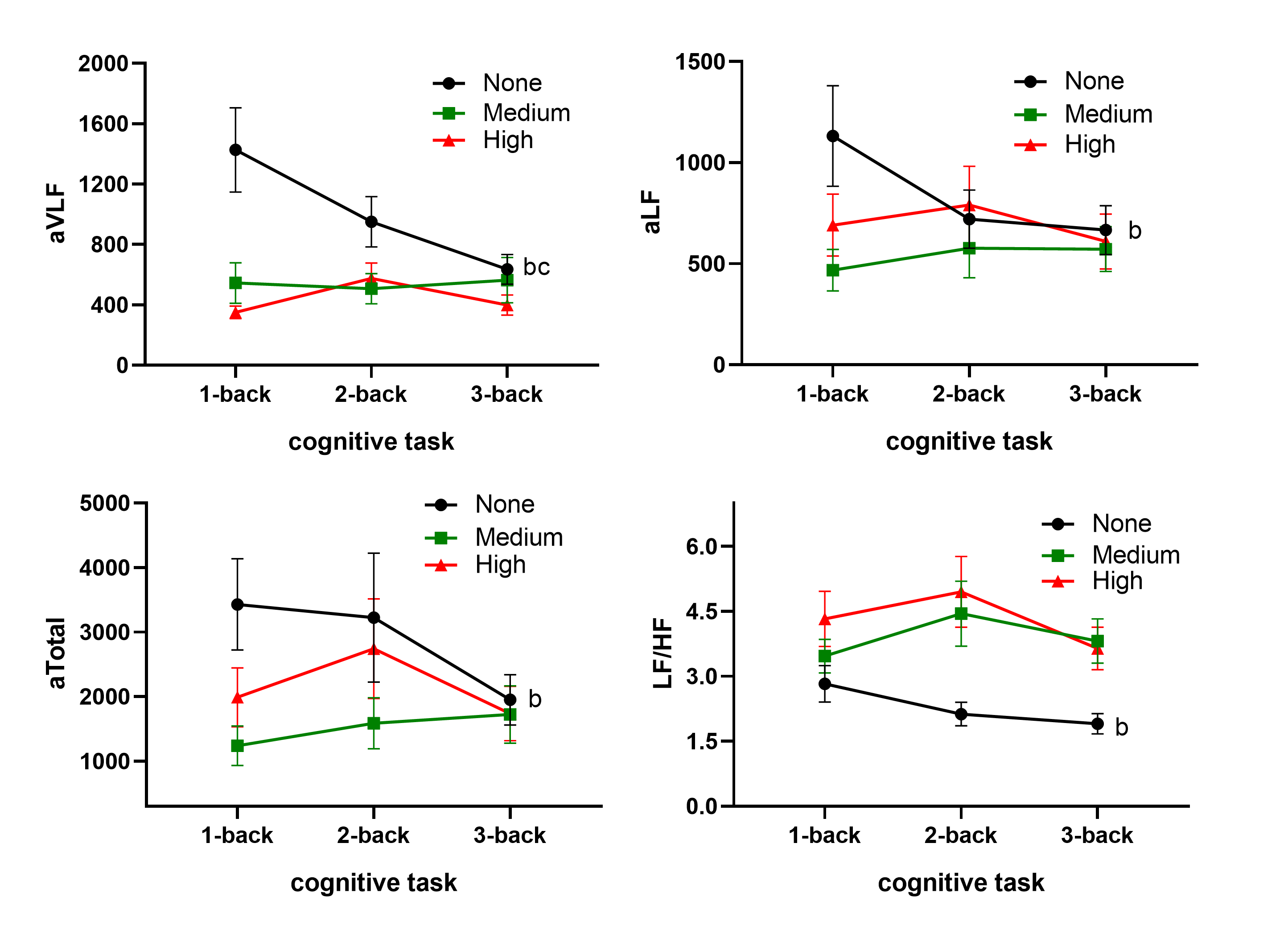


**FIGURE S4**

Influence of CL change on HRV when accompanied by PL. Data are plotted as the mean ± standard error. aLF, absolute value of power density in low frequency band; aTotal, absolute value of total power density; aVLF, absolute value of power density in very low frequency band; CL, cognitive load; LF/HF, ratio of low-frequency and high frequency power; PL, physical load.

**2 Similar HRV baseline**

According to the results presented in **Table S1**, there was no significant difference in HRV parameters between different PL before cognitive tasks (*P* > 0.05). The results suggested that the activity of the autonomic nervous system in different PL groups was consistent in the resting state.

**TABLE S1** Comparison of HRV at baseline under different PL conditions.

| HRV | **None PL** | **Medium PL** | **High PL** | *χ^2^* | *P*-value |
| --- | --- | --- | --- | --- | --- |
| SDNN | 79.00 (68.83) | 65.80 (52.55) | 66.50 (98.23) | 1.688 | 0.430 |
| NN50 | 83.00 (102.75) | 68.50 (106.50) | 91.50 (99.75) | 1.168 | 0.558 |
| pNN50 | 27.90 (41.23) | 22.20 (37.93) | 22.80 (41.55) | 2.315 | 0.314 |
| RMSSD | 48.25 (104.43) | 43.70 (51.73) | 40.75 (101.20) | 1.313 | 0.519 |
| MeanHR | 83.65 (23.05) | 81.35 (23.18) | 79.10 (21.43) | 1.000 | 0.607 |
| aVLF | 824.42 (1,541.72) | 474.10 (763.99) | 583.19 (913.49) | 2.250 | 0.325 |
| aLF | 623.43 (1,376.89) | 526.33 (678.76) | 477.26 (1,384.05) | 1.188 | 0.552 |
| aHF | 387.08 (1,676.21) | 260.90 (788.40) | 218.32 (1,674.15) | 1.313 | 0.519 |
| aTotal | 1,932.75 (3,568.60) | 1,524.08 (2,952.17) | 1,662.93 (5,057.39) | 3.563 | 0.168 |
| LF/HF | 1.44 (1.80) | 1.46 (2.41) | 1.72 (2.12) | 4.188 | 0.123 |
| nHF | 41.00 (31.10) | 40.75 (24.03) | 36.70 (30.95) | 4.188 | 0.123 |
| nLF | 59.00 (31.10) | 59.25 (24.03) | 63.30 (30.95) | 4.188 | 0.123 |

Note: HRV parameters did not conform to a normal distribution and homogeneity of variance; therefore Friedman’s test was used. The statistic is the “*χ^2^*” value. Data are presented as median and interquartile range.

aHF, absolute value of power density in high frequency band; aLF, absolute value of power density in low frequency band; aTotal, absolute value of total power density; aVLF, absolute value of power density in very low frequency band; LF/HF, ratio of low-frequency and high frequency power; meanHR, mean heart rate; nHF, normalized high frequency power; nLF, normalized low-frequency power; NN50, number and percentage of the difference between adjacent RR intervals > 50 ms; PL, physical load; pNN50, percentage of the difference between adjacent RR intervals > 50 ms; RMSSD, root mean square difference; SDNN, standard deviation of normal to normal beats.
